# Demographic, socio-economic and other associated risk factors for self-medication behaviour among university students of Sri Lanka: a cross sectional study

**DOI:** 10.1186/s12889-020-08622-8

**Published:** 2020-05-04

**Authors:** Nirma Subashini, Lahiru Udayanga

**Affiliations:** 1 Department of Horticulture & Landscape Gardening, Faculty of Agriculture & Plantation Management, Makadura, Wayamba University of Sri Lanka, Gonawila, Sri Lanka; 2 Department of Biosystems Engineering, Faculty of Agriculture & Plantation Management, Makadura, Wayamba University of Sri Lanka, Gonawila, Sri Lanka

**Keywords:** Self-medication, Knowledge attitudes and practices, Risk factors, Undergraduates, Sri Lanka

## Abstract

**Background:**

Self-prescribing practices are considered as a significant issue in the health sector due to mal-practices. This has become a more worsen issue in developing countries with easy access to medication. Current study was undertaken to estimate the prevalence of self-medication and to evaluate the driving factors behind this behavior, among university students of Sri Lanka.

**Method:**

A total of 700 randomly selected undergraduates of three state universities in Sri Lanka, were recruited as the study population for the cross-sectional study. Information on socio-demographic, Knowledge, Attitudes and Practices relevant to Self-Medication (SM) were gathered using an interviewer administered questionnaire. Binary logistic regression was used to calculate the Odds Ratios (OR) and the 95% confidence intervals of the OR for socio-demographic risk factors on SM.

**Results:**

Around 78% of the studied population denoted a SM behaviour. Only, 37.7% of respondents were familiar with the classification of “Over the Counter” (OTC) drugs, while majority were well aware of the risks of SM (> 50%). Fever (61.3%) and cough (56.7%) were the major health issues for SM, while antipyretics and drugs for cough and runny nose (73.6%) were the mostly used medication. Previous experience (76%) and trivial nature of health issues (73%) were recognized as the major reasons for SM. Majority of respondents declared that community pharmacies (86.9%) and left over medication from previous prescriptions (51%) were the sources for SM. Based on the binary logistic regression, age, residence locality, field of study and academic year were recognized as significant risk factors (*P < 0.05*) for SM within the studied undergraduate population.

**Conclusion:**

Based on the high prevalence rate of SM, the health authorities of Sri Lanka should pay more attention towards the wellbeing and responsible medication usage of undergraduates. Designing of effective tools and regulations to monitor the selling of medication and improving the health education are recommended to ensure responsible SM within the country.

## Background

Use of medications for treating themselves, has remained as a common inherent tendency of humans since the initiation of great civilizations. People, belonging to different tribes/civilizations, have practiced self-care measures to maintain their own health through Self-Medication (SM). This involves quick and direct admittance to treatment in relieving minor ailments [1, 2]. SM refers to the use of nonprescription medicines (including herbal/traditional products and Over the Counter[OTC] drugs) to treat self-recognized minor ailments, without any consultation of a medical practitioner [2]. Obtaining medication without a prescription, sharing medications with others, or utilizing a medication that is already available in the residence (including leftovers from previous prescriptions)come under SM [1, 3].

The patterns of medication is an important health indicator, which reflect the prevalence of diseases and the degree of therapeutic resource utilization within a community [4]. Therefore, SM is often used as a significant indicator in evaluating the effectiveness of the health sector [1, 4, 5]. If done appropriately, the responsible form of SM can save time for the consultation of the medical practitioner and address the limitations in availability of medical practitioners [2, 6, 7]. Further, it may be economical against minor ailments as well as for acute conditions at an urgent situation [7, 8]. Despite, allowing the patients/consumer to play an active role in his/her own care [9], inappropriate form of SM is a major constraint in ensuring the safe and effective use of essential medicines [4, 5]. Expansions in the availability of pharmaceuticals, in terms of quantities and varieties, at the global scale has resulted easy accessibility for people leading to elevated rates of misusing medicines [10]. This in turn leads to health risks and cause severe economic burdens to governments, while influencing the affordability of medicine by the patients [9–11]. Antibiotic resistance, treatment failures, drug toxicities, adverse drug reactions and prolonged suffering are some of the significant adverse effects of inappropriate use of medicine through SM [6–9]. It may also lead to increased dependency on drugs and deterioration in health status, due to delays in seeking medical advice, when needed [4, 9, 11].

Increasing rates of antibiotic resistance has become the most serious issue arising due to inappropriate use of antibiotics in SM [12]. According to the World Health Organization (WHO), sub-optimal prescribing practices such as inadequate dosing, incomplete treatment courses and indiscriminate drug use arising due to SM have contributed to the emergence and spread of antimicrobial resistance and emergence of multi-drug resistant pathogens [10, 13]. Therefore, absurd use of antibiotics and other medicines could directly lead to wastage of medical resources, while challenging the safe and efficacious use of medicines [14–16].

Consequently, there is a growing concern on the illogical use of medicines among the professionals, resulted due to the increase in SM at the global scenario [17]. The prevalence of SM has been estimated to be in-between 10.3 to 87.0% worldwide. A rate of 68% is reported in the European region, while developing countries tend to report further elevated rates of SM that may reach up to 92% [9, 16, 18].Higher rates of SM have been reported among the university students in regional countries such as Bangladesh, Pakistan and Saudi Arabia, mostly due to academic competition and pressure [6, 19–22]. Despite elevated prevalence of SM, the overall knowledge of patients and common public on the potential risks of SM remains to be poor, even with prescribed medication for chronic conditions [4, 9]. Majority tend to engage in SM practices solely due to the perceived benefits such as quick relief, for economic benefits, to save time, lack of availability of healthcare personnel and to avoid public embarrassment [2, 4, 5, 22]. On the contrary, less attention is paid towards potential side-effects of SM [4], making SM a serious health concern in many countries.

Recent studies have suggested that the misuse of nonprescription drugs amongst adolescents is growing alarmingly. The high involvement of the present day youth with media and internet has increased their exposure to advertising on pharmaceuticals, thereby motivating them towards SM [18, 23]. According to few recent studies conducted in Jordan [24] and Saudi Arabia [25, 26], medical websites and television or radio advertisements may play a notable role as knowledge sources of SM among undergraduates. On the other hand, a notable fraction of the youth community will be future health care providers, whose own health care seeking behavior would influence their future medical practices [18]. This has raised critical concerns on incorrect self-diagnosis, drug interaction, and drug usage other than for the original indication [23]. Neglectful regulatory systems in developing countries often tend to contribute towards high prevalence rates of SM [20]. Meanwhile, strict regulations on usage of medicines have suppressed illogical use of SM [14, 27, 28]. Furthermore, drugs that essentially require prescriptions for purchase in developed countries are often available as OTC in developing countries, elevating the potential risk of SM on the health sector [29]. Thus, many developing countries have become ideal breeding grounds for drug-resistance and drug addictions, due to poor supervision of prescriptions [30].

Accordingly, SM has become a common practice in many of the Asian countries including Sri Lanka. Available studies in Sri Lanka has revealed that, majority of common diseases such as cold (68.2%), sore throat (52.7%) and fever (31.0%) are being treated through SM. Previous prescriptions for similar symptoms or pharmacists opinion towards the illness have been found to be the driving factors for such behaviours [31–33]. SM is recognized to be influenced by a variety of demographic (education level, economic power, family background, knowledge and attitudes on SM), cultural (religious beliefs) and country specific (existing regulations, nature of the health care system and market policies on medication etc.) factors [5, 12, 34, 35]. Evaluation of the potential risk factors that motivate a society towards SM, is of immense importance in guiding the policy makers to control ill effects of SM in developing countries [36].

Despite the critical importance of self-prescribing practices for the health and well-beingof the communities in developing countries, limited focus has been paid to investigate the prevalence of SM. Further, the critical risk factors influencing SM in Sri Lanka remain poorly investigated. Most of the previous studies have only concentrated on the SM practices among athletes, maternal and selected urban populations within Sri Lanka. Meanwhile, SM practices among the university student communities that has become a priority area at the global context remains poorly studied [37, 38]. Therefore, the current study was conducted to evaluate the knowledge, attitudes/perceptions and behaviors/practices on SM and to characterize the critical risk factors influencing the SM behaviour among adolescent undergraduates in the university system of Sri Lanka.

## Method

### Selection of study population

An analytical cross-sectional survey was conducted from February to April 2019.A total of700 randomly selected undergraduates of three state universities in Sri Lanka, namely University of Moratuwa, University of Kelaniya and Wayamba University of Sri Lanka, were recruited as the study population by following Krejcie and Morgan [39]. During the calculation of sample size, it was assumed that the marginal error is 3.5%, population proportion is 0.5, while the actual population size of the whole undergraduate community per year is 30, 000. During the fieldwork, the sample size was increased to reach 700 undergraduates. Any student who refused to participate in the study due to one or more reasons such as religious beliefs or an opinion that it is not worth participating, were not considered for the survey. New members were recruited, randomly to cater for such exclusions. The selection of students was done in such a way to ensure effective representation of different streams of study and levels of undergraduates.

### Data collection

The socio-economic, Knowledge, Attitudes and Practices (KAPs) of the selected undergraduates on self-medication behavior were collected using an interviewer-administered questionnaire prepared in three local languages (Sinhala, English and Tamil). The selected undergraduates were interviewed by a group of 5 trained interviewers.

The questionnaire covered the following areas: 1.Socio-demographic information (age, gender, nature of the residence locality, field of study and the level of study of the undergraduates); 2.Self-medication and associated practices (practicing of SM, frequency of SM, maximum number of medications used at once, symptoms for which SM is practiced); 3.Knowledgeon SM (knowledge on Over the Counter Drugs (OTC) and the possible risks of SM); 4 Attitudes towards SM (Reasons for SM, factors considered during selection of medication, Preferred sources of information and medication purchase). Prepared questionnaire was pre-tested and validated by using a preliminary sample of 30 undergraduates.

### Data interpretation and statistical analysis

All collected data were double-checked and verified on the same day for completeness and consistency, prior entering into Microsoft Access® data sheets (Version, 2013). Quality controlling procedures were followed throughout the process by trained personnel. Meanwhile the accuracy of data were routinely checked by cross tabulations and logical checks. Discrepant data were checked against original data forms and any mistakes were promptly corrected. The binary logistic regression approach was used to calculate the Odds Ratio (OR) and the 95% Confidence Intervals (CI) of the OR for socio-demographic risk factors on SM. All statistical analysis were done using the SPSS package 23.

## Results

### Demographic and socio-economic factors

Among the 700 respondents, majority (55.1%) were males, while the age group of 24 to 26 years dominated accounting for 54.9% of the study population. In case of locality, a relatively higher portion of respondents (41.5%) were residing in semi-urban areas followed by urban (32.1%) and rural (26.4%) areas. All the respondents had diverse fields of study, where majority studied agriculture and food sciences (30.3%), followed by biological sciences (16.3%) and technology stream (15.1%). Meanwhile, fourth year undergraduates dominated within the study population (29.1%), while first year undergraduates denoted the least frequency of 20.6% (Table 1).

**Table 1:** Demographic and socio-economic factors of the study population

Parameter		Total respondents	
		n	%
Gender	Male	386	55.1
	Female	314	44.9
Age (Years)	20–23	280	40.0
	24–26	384	54.9
	> 26	36	5.1
Locality	Rural	185	26.4
	Semi-Urban	290	41.5
	Urban	225	32.1
Field of study	Agriculture & Food Sciences	212	30.3
	Technology	106	15.1
	Biological Sciences	114	16.3
	Commerce and Management	76	10.9
	Physical and Applied Sciences	92	13.1
	Arts	100	14.3
Year of study	First year	144	20.6
	Second year	170	24.3
	Third year	182	26.0
	Fourth year	204	29.1

### Knowledge on self-medication (SM)

Of the respondents, 62.3% were not aware of the classification of OTC medications, which can be purchased without any prescription. Meanwhile 86.0% were knowing what antibiotics are. Surprisingly, only 54.9% of the study population correctly mentioned that antibiotics are used against bacterial infections, while a considerable fraction of undergraduates had a limited knowledge on this (45.1%). Relatively higher percentage of undergraduates were well aware that humans can become resistant to antibiotics (65.9%) and the possible side effects of strong medications (76.4%). Around 77% of the respondents knew that prior knowledge on the mechanism of drugs is necessary before initiating SM practices. However, a notable fraction was not aware of the facts such as higher doses not always result fast recovery (35%), low doses can’t always prevent less adverse conditions (22%) and medication can’t be stopped when basic symptoms fade away (21%).

Limited knowledge in medicine (64.0%), risk of having adverse reactions/effects (60.4%), risk of misdiagnosing (53.7%) and misuse of drugs/medications (49.6%) were recognized as the major risks associated with SM. The proportion of respondents aware of possible risks such as the development of drug/medication dependence and administration of medications wrongly (wrong doses and inappropriate methods etc.) were relatively low (24.3 and 27.1%, respectively). Even though, only a limited faction of study population (9.1%) was knowing the chemical composition of drugs used by them, approximately quarter of the respondents (26.3%) were capable of fully understanding the instructions that comes with medication. Meanwhile, 54.7% of respondents were proficient in partially understanding the instructions (Table 2).

**Table 2:** Knowledge of the study population on medical drugs and Self-Medication

Parameter		Total respondents	
		n	%
Familiarity with the classification of OTC	Yes	264	37.7
	No	436	62.3
Do you know what antibiotics are?	Yes	602	86.0
	No	98	14.0
Antibiotics are used for?	Virus infections	313	44.7
	Bacterial infections	384	54.9
	Other	3	0.4
Risks associated with Self-Medication are?	Risk of using wrong drugs	347	49.6
	Lack of knowledge about medicines	448	64.0
	Risk of having adverse effects	423	60.4
	Risk of misdiagnosing	376	53.7
	Administrating medications wrongly	260	27.1
	Risk of drug dependence	170	24.3
	Other	10	1.4
Which of the following statement(s) about antibiotics is (are) correct?	Higher doses always result fast recovery	245	35.0
	Low doses can always result less adverse conditions	154	22.0
	Switching drugs always enhance drug effect	52	7.4
	Switching drugs always reduce adverse reactions	40	5.7
	A prior knowledge on the mechanism of drugs is necessary before initiating self-medication practices	539	77.0
	Medication can be stopped when basic symptoms fade away	147	21.0
Can humans become resistant to antibiotics?	Yes	461	65.9
	No	239	34.1
Can Self-Medication cause side effects?	Yes	535	76.4
	No	165	23.6
Do you know the chemical composition of the drugs that you are using?	Yes	64	9.1
	No	636	90.9
Level of understanding of the instructions used for Self-Medication	Fully understood	184	26.3
	Partially understood	383	54.7
	Did not understand	133	19.0

### Practices on self-medication (SM)

Of the entire study population, 546 undergraduates (78.0%) practiced SM. Majority (47.1%) of the undergraduates were practicing SM more than 5 times per year, followed by once in every two three months (31.1%). Only, 2.3% of the respondents were frequently indicating SM practices at a weekly interval (Table 3). Approximately 52.9% of the respondents were using a single drug at a time, while the proportion of undergraduates that used multiple drugs were least (18.9%). Among the respondents, only 63.3% were occasionally changing the administrated dose of SM. Interestingly, 21.3% were never changing the initial dosage. On the other hand, 15.4% were always changing the administrated dosage of medication. Unsatisfaction of the administering dosage to cure the disease (36.7%), worsening of the existing condition (23.9%) and improving of the existing condition (21.1%) were the major reasons for changing the dosage during SM.

**Table 3:** Practices of the study population related to Self-Medication

Parameter		Total respondents	
		n	%
Practicing of Self-Medication	Yes	546	78.0
	No	154	22.0
Frequency of Self-Medication	Once a week	16	2.3
	Every 2–3 weeks	48	6.9
	Once a month	88	12.6
	Every two three months	218	31.1
	More than 5 times per year	330	47.1
Number of medicines simultaneously used	1	370	52.9
	2	198	28.3
	Multiple	132	18.9
How often do you change the dosage?	Always	108	15.4
	Occasionally	443	63.3
	Never	149	21.3
Reasons for changing the dosage	Improving conditions	148	21.1
	Worsening conditions	167	23.9
	To reduce adverse conditions	128	18.3
	Felt insufficient to cure the cause	257	36.7
Symptoms for Self Medication	Cough	397	56.7
	Stomachache	297	42.4
	Fever	429	61.3
	Skin wounds/symptoms	157	22.4
	Allergies	97	13.9
	Eye symptoms	4	0.6
	Ear symptoms	14	2.0
	Diarrhea	58	8.3
	Back pain relief	112	16.0
	gastritis	325	46.4
	Other (Specify)	122	17.4
Common drugs (medicines) used	Antipyretics for fever: Paracetamol	515	73.6
	Pain killers: Paracetamol, Panadine, Aspirin, Brufen	372	53.1
	Drugs for cough/runny nose: Cough syrups, piriton, cetirizine	515	73.6
	Laxatives: Lactulose	19	2.7
	Vitamins: Vitamin C, Vitamin E, Vitamin B, Iron, Folic acid, Calcium	283	40.4
	Antiallergic: Fexofenadine, Prednisolon, Piriton	126	18.0
	Sleeping pills	14	2.0
	Antibiotics: amoxicillin, cloxacillin	272	38.8
	Pain relief balms: Diclofenac gel	301	43.0
	Inhalers	50	7.1
	Cetrazine	332	47.4
	Drugs for gastritis	290	41.4
	Anti-itching balms: Lactocalamine, hydrocortisone	165	23.6
	Diarreha: Jeevani	101	14.4
	Other	4	0.6
Determining the dose	Checking the package	485	69.3
	Consulting a pharmacist	420	60.0
	Consulting family members/friends	265	37.9
	From my previous experience	369	52.7
	By guessing the dosage	33	4.7
	Consulting a medical officer	89	12.7
How often do you check the instructions for drug usage?	Always	205	29.3
	Occasionally	308	44.0
	Never	187	26.7
Do you keep old medicine/left overs at your home?	Yes	483	69
	No	217	31
Have you ever found out that you had taken the same drug with different names at the same time?	Yes	205	29.3
	No	485	69.3
Have you faced any side effects due to Self-Medication?	Yes	281	40.1
	No	419	59.9
What did you do when side effects arise due to Self-Medication?	Switch to another drug	58	8.3
	Consulted pharmacy staff	318	45.4
	Consult family member/friend	107	39.1
	Consulted a physician	46	6.6
	Other (specify)	4	0.6

Fever, was the major cause for practicing SM (61.3%) followed by cough, gastritis and stomachache (56.7, 46.4 and 42.4%, respectively). Interestingly, complications in eyes (0.6%) and ears (2%) were the least contributing causes for SM within the study population. Antipyretics for fever (73.6%), drugs for cough/runny nose (73.6%), pain killers (53.1%) and Cetrazine (47.4%) were the most consumed medications used for Self-Medication by the undergraduates (Table 3). On the contrary, other medications such as traditional medicines, Ayurvedic products, primary wound care medication (0.6%) and sleeping pills (2%) were the least used medications by the respondents.

Checking the package (69.3%) and consulting a pharmacist (60%) were the most practiced methods of dose determination during SM. Among them, only 12.7% undergraduates were consulting a medical officer for accurate instructions. Interestingly, 29.3% of the respondents were always checking the instructions of the medicines that they consume, while 44.0% were checking the instructions occasionally. Meanwhile, a notably high fraction (26.7%) were never inspecting the safety instructions for drug usage. A total of 483 undergraduates (69%) of the study population were keeping the left overs of previously used medications at home, which could be a contributing factor for SM (Table 3). Approximately 29.3% of the respondents accepted that they might have taken the same medication with different brand names at the same time. Meanwhile 40.1% stated that they have faced adverse impacts of SM. Consulting the pharmacy staff (45.4%) and family member/friend (39.1%) were the mostly practiced response for such adverse reactions (Table 3).

### Attitudes on self-medication (SM)

Previous experience (76.0%), trivial nature of issues (73.0%)and necessity of avoiding embarrassing moments (52.6%) were the major contributing factors that encouraged the respondents for SM. Meanwhile, time, urgency and economic constraints were having limited influences (23.0, 22.7 and 17.4%, respectively) on SM behaviour. A higher proportion of the study population (40.7%) were satisfied with any medication provided by the pharmacist. Interestingly, a notable percentage of respondents were paying attention to the possible side effects (32.1%), brand name (31.7%) and price (25.7%), when purchasing drugs or medication. Community pharmacies (86.9%) and left over from previous prescriptions (51%) were the most preferred sources of obtaining medications. On the contrary, friends (21.1%) and neighbours (4.1%) were the least preferred sources (Table 4).

**Table 4:** Attitudes of the study population on Self-Medication

Parameter		Total respondents	
		%	n
Reasons for Self-Medication	Previous experience	532	76.0
	Problem too trivial (minor)	511	73.0
	Urgency of the problem	159	22.7
	No time to go for a doctor	161	23.0
	Consulting a doctor is too expensive	122	17.4
	Quick relief	279	39.9
	To avoid the embarrassing moments	368	52.6
	Other (specify)	3	0.4
Considerations during purchase	Type of the drug	89	12.7
	Brand name	222	31.7
	Price	180	25.7
	Side effects	225	32.1
	Anything given by the pharmacist	285	40.7
Preferred sources of medication purchase	Community pharmacies	608	86.9
	Left over from previous prescriptions	357	51.0
	Family members	250	35.7
	From friends	148	21.1
	Neighbours	29	4.1
Preferred sources of information for Self-Medication	Previous doctor’s prescription	480	68.6
	Opinion of a friend	240	34.3
	Own previous experience	545	77.9
	Pharmacist advise	335	47.9
	Opinion of family member	318	45.4
	Advertisements/Leaflets	79	11.3
	Internet and social media	257	36.7
	Other (Specify)	0	0.0
Do you believe that you might have taken counterfeit drugs?	Yes	143	20.4
	No	557	79.6
Can common diseases be successfully treated through Self-Medication?	Yes I can	324	46.7
	Not sure	308	44.0
	No, I cannot	68	9.7
Do you recommend drugs (medicines) for others?	Yes	305	43.6
	No	395	56.4

Own previous experience (77.9%) was the most preferred source of advice for selection of medications, followed by a previous prescription of a doctor (68.6%), pharmacist advice (47.9%) and opinion of a family member (45.4%). It was interesting to note that internet and social media accounted for around 36.7% preference among the undergraduates as a source of information. A majority of respondents believed that they have not consumed any counterfeit drugs (79.6%). At the same time, higher percentage of respondents (53.7%) were not sure whether common disease conditions can be cured by SM. On the contrary, only 46.7% believed that common diseases can be successfully treated via SM. Interestingly, a notable fraction of undergraduates (43.6%) were willing to recommend medicines for their friends or family members, which could promote SM (Table 4).

### Risk factors for self-medication

As suggested by the results of binary logistic regression, all the basic demographic and socio-economic factors (except for gender) were having a significant association with the practicing of SM by the studied population (Table 5). Even though, males were having a relatively higher preference for SM (*P* = 0.08; OR = 1.65; CI = 0.92–2.01) than the females, the association of gender and SM behavior was non-significant. The highest prevalence of SM was observed among the students belonging to the age group of 24–26 years (81.3%; OR = 1.23), followed by 20–23 (77.9%; OR = 1). Students from urban (92.4%; OR = 10.18; CI = 8.20–10.75) and semi-urban (81.7%; OR = 3.72; CI = 2.60–4.13) areas had significantly higher tendency (*P* = 0.004) for SM behaviour, than those from rural localities (54.6%). Interestingly, undergraduates from Biological Sciences (94.7%; OR = 2.08; CI = 1.46–3.02) showed a significantly higher prevalence of SM, followed by undergraduates from Agriculture & Food Sciences (89.6%; OR = 1). In case of academic year of study, the final (fourth) year undergraduates denoted the highest prevalence of SM (97.1%; OR = 23.57; CI = 20.88–24.45), followed by third year undergraduates (85.7%; OR = 4.29; CI = 3.05–4.82). The tendency for practicing SM significantly increased with the academic year (*P* < 0.001) as shown in Table 5.

**Table 5:** Results of the binary logistic regression

Parameter		Total respondents		Prevalence of SM behaviour		*P* value	Odds Ratio	95% Confidence Interval for the Odds ratio Number
		n	%	n	%			
Gender	Female	314	44.9	230	73.2	0.081	Reference	
	Male	386	55.1	316	81.9		1.65	0.92–2.01
Age (Years)	20–23	280	40.0	218.0	77.9	0.001	Reference	
	24–26	384	54.9	312.0	81.3		1.23	1.05–1.61
	> 26	36	5.1	16.0	44.4		0.23	0.12–0.74
Locality	Rural	185	26.4	101.0	54.6	0.004	Reference	
	Semi-Urban	290	41.4	237.0	81.7		3.72	2.60–4.13
	Urban	225	32.1	208.0	92.4		10.18	8.20–10.75
Field of study	Agriculture & Food Sciences	212	30.3	190.0	89.62	< 0.001	Reference	
	Arts	100	14.3	41.0	41.00		0.08	0.03–0.68
	Biological Sciences	114	16.3	108.0	94.74		2.08	1.46–3.02
	Commerce and Management	76	10.9	52.0	68.42		0.25	0.13–0.71
	Physical and Applied Sciences	92	13.1	70.0	76.09		0.37	0.22–0.97
	Technology	106	15.1	85.0	80.19		0.47	0.27–0.94
Year of study	First year	144	20.6	84.0	58.3	< 0.001	Reference	
	Second year	170	24.3	108.0	63.5		1.24	1.06–1.70
	Third year	182	26.0	156.0	85.7		4.29	3.05–4.82
	Fourth year	204	29.1	198.0	97.1		23.57	20.88–24.45

## Discussion

### Practices on SM

SM is an important aspect, which should be rigorously monitored by the health authorities (especially in developing countries) to ensure safe and responsible usage of drugs by patients. This requires effort to aware the patients with adequate information regarding the usage of pharmaceuticals and instances to consult a qualified medical officer, when needed [24, 40, 41]. The undergraduate population in the current study reported a 78% prevalence of SM at higher SM frequencies such as 2–3 times per month or more (52.9%). Several studies conducted in Oman (94%), Palestine (98%), India (87%), Pakistan (76%)also have reported similarly high levels of SM among undergraduates [18, 23, 42–44]. On the contrary, Buke et al. [37] has reported only a 45% prevalence of SM among university students in Turkey. Meanwhile a Brazilian study conducted with university healthcare and non-healthcare students has shown 57.7% prevalence of SM [45]. Findings of the current study stands in line with few similar studies conducted with university students in Pakistan [22], Slovenia [46] and Palestine [18].

Ear and eye related issues were the least dealt issues through SM, mostly due to the higher sensitivity of those systems. Antipyretics (such as Panadol, Paracetamol), drugs for cough/runny nose (cough syrups, piriton, cetirizine) and pain killers (Paracetamol, Panadine, Aspirin and Brufen) were the mostly used medication that denoted a usage by more than 50% of the studied population. Several recent studies have reported similar findings, where painkillers, flu/cough remedies have emerged as most commonly used medication in SM [2, 5, 8, 17, 32, 45]. Especially, few studies that focused on the SM behaviours of undergraduate populations in Bangladesh [21] and Jordan [24] have also reported a similar observation in the region. Even though, a higher usage level of antibiotics have been reported in many previous studies [12, 17], only 38.8% of the entire study population administrated antibiotics during SM. A similar rate of antibiotic usage has been found among medical and non-medical students in Jordan [24].

### Knowledge on SM

Despite the high rates of SM, only approximately one third of the entire study population (37.7%) was familiar with the classification of OTC drugs. This suggested that SM is practiced with limited knowledge. Further, only 53.3% of the study population were truly aware of the fact that antibiotics are used against bacterial infections, among 86% who claimed to have knowledge on antibiotics. A notable fraction of the study population carried misconceptions on antibiotics such as higher doses always result fast recovery (35%), low doses can always result less adverse conditions (22%) and administration of antibiotics can be stopped immediately when basic symptoms fade away (21%). A recent study conducted by Grigoryan *et al.* has also reported that people in Southern and Eastern European countries tend to possess a less accurate knowledge on antibiotics, especially regarding the effectiveness of antibiotics against bacterial and viral infections [38]. However, majority (65.9%) of the respondents were aware of antibiotic resistance and the ability of SM causing ill effects (76.4%). This level of understanding within undergraduates of Sri Lanka remains higher than in Europe (48%), while lies closer (73%) to the awareness level in United States [38, 47]. A recent study conducted in Turkey has also reported that a high proportion of undergraduates tend to engage in SM, since they only know that SM is wrong and is capable of causing ill effects [37]. Regardless of this primary understanding, more awareness on the devastating ill effects of SM should be communicated to the undergraduates to promote responsible SM within Sri Lanka. Few recent studies that have focused on the undergraduate communities in several regional countries have also recommended such measures to enhance the responsible SM [7, 21, 24, 26].

High SM prevalence within the studied university population, despite the reasonable knowledge on the risks of SM such as risk of using wrong drugs (49.6%), lack of knowledge about medicines (64%), risk of having adverse effects (60.4%) and risk of misdiagnosis (53.7%), agrees with the findings of previous studies [4, 24, 48]. This has been described as a phenomenon, whereby the practitioners of SM tend to outweigh the potential risks of side-effects with potential benefits, due to their belief that only safe medicines are permitted to be sold as OTC [17, 48]. Further, the limited understanding on the classification of OTC and malpractices of pharmacists (who sell non-OTC medication without proper prescriptions) could also be prominent risk factors for SM prevalence [12, 23, 24].

### Attitudes on SM

The risk of SM behavior among the studied Sri Lankan university population further increased since the preferred methods to determine the administration dosage of medication were checking the package, consulting a pharmacist or based on previous experience. Therefore, administration of appropriate doses remains questionable as majority of the respondents claimed that they are capable of understanding the instructions that comes with the package, partially (54.7%) or very poorly (19%). A recent study in China has also reported a similar low level of understanding the instructions that comes with medication among the university students [17]. Majority of students changed the administration dosage occasionally (63.3%) or never changed the dosage (21.3%), which could result severe side effects in case of misdiagnosis or due to over use [2, 12]. Irrational use of antibiotics arising due to inappropriate antibiotic prescribing, selection of wrong drug, dosage and inappropriate period of use could lead to serious health concerns and antibiotic resistance [12, 49, 50].

More than one third of the study population have faced side effects of SM previously and stop taking the drug (76%) or consulting a pharmacist (45.4%) has remained as their preferred option. Meanwhile only a limited number of respondents tend to consult a physician in such occasions. This behavior could also worsen the risk of SM on the well-being of the community, due to poor treatment seeking behavior [12, 24]. Previous experience, trivial nature of the problem, need for avoiding the embarrassing moments and quick relief have been the major reasons for SM among the studied university student population. These findings agree with many previous studies on SM conducted among different populations [2, 20–26, 51]. Even though, majority of the respondents were accepting any medication provided by the pharmacist, a notable fraction were considering the potential side effects and the brand name they preferred in purchasing medication. A study conducted in Saudi Arabia has reported that majority (84.1%) pay attention to the effectiveness of medications, followed by the brand [51].

Community pharmacies (86.9%) was the most preferred source of medication purchase for SM followed by left over from previous prescriptions and family members. Community pharmacies often remain as the most preferred source of medication and other therapeutic drugs in many studies conducted all over the world [12, 20, 24, 40, 52, 53]. Availability of left-over medication or home pharmacies could provide free access and easy visualization of medication, which in turn act as promoters of SM [2, 20, 54]. As emphasized by Reynolds and McKee left over medication from previous prescriptions suggest that either doctors over-prescribe and/or the patients not complying with the prescription [55].

Own previous experience (77.9%), previous prescriptions of a doctor (68.6%), pharmacist advice (47.9%) and opinions of family members (45.4%) were used for the decision making in SM in the current study. Several previous studies have also reported similar findings [2, 18, 20–24, 44, 56]. Both family members and the pharmacists often tend have inadequate knowledge about antibiotics [2, 40, 56]. Further, previous prescriptions may not be ideal for the current scenario, elevating the risk for unintended side effects due to SM. Continuous use of the same medicines (based on previous prescriptions) could lead to development of drug resistance, addition and concealing of malicious and potentially fatal diseases [17, 57–59]. In addition, following of an advice of a family member in selecting medication would evidence for culturally inherited acceptance of self-medication [40, 55, 56], which remain as a critical risk factor.

Majority of respondents believed that common diseases could be successfully treated through SM, which remains as a crucial factor to be considered by the health authorities in the country [7, 8, 24]. Further, nearly half of the respondents (43.6%), accepted that they are recommending drugs for their friends and other family members, which is a serious concern. On the contrary, a recent study conducted in Brazil and Saudi Arabia have reported that a notable fraction of university students tend to discourage SM practices among their friends and relatives [2, 20].

### Risk factors for SM

Even though males were having a higher tendency for SM (OR = 1.65) in comparison to female undergraduates, the effect of gender on SM behavior was found to be insignificant. This finding agrees with several previous studies conducted in India [7], Lithuania [12] and western China [40], while an opposite trend has been reported from Brazil [2].Few recent studies conducted in Bangladesh [21] and Saudi Arabia [25] also reported that the effect of gender on SM remained insignificant among undergraduates. Age was also a significant risk factor, where younger students showed a higher prevalence rate of SM, which agrees with several other studies [2, 17, 38]. Students from urban and semi-urban localities denoted significantly higher prevalence rates of SM, suggesting that locality is a critical risk factor. Undergraduates from such areas have more exposure to medical services and media, which directly encourage them for SM [18, 23]. Further, the availability of community pharmacies is also high in such areas, unlike in rural areas, which could have resulted the above observation [23]. On the contrary, an opposite trend has been reported in Lithuania, where rural people have shown the high prevalence of SM behaviour [12].

Undergraduates from science background (biological sciences and agriculture & food sciences) denoted higher SM bahaviours than undergraduates from art or commerce fields. Undergraduates with a science background may have a better knowledge on medication, leading to elevated SM rates than undergraduates from non-science backgrounds [24, 26]. Academic year of study also emerged as a significant risk factor, where final year students (third year and fourth year) had higher prevalence rates of SM. However, a previous study conducted in Sri Lanka has reported a similar positive trend among the academic year of students and the SM behaviour of undergraduates in the University of Peradeniya, which was not statically significant [32]. Few regional studies conducted in Pakistan [19] and Saudi Arabia [26] have evidenced that SM practices have increased with the academic year in undergraduate populations. An opposite trend has been reported in Brazil, where final year students have shown lesser SM preference, which has also remained non-significant [2]. Similar reluctance in SM has been shown by a cohort of pharmacy and medicine undergraduates in Saudi Arabia with the progression in educational career [20]. However, the influence of friends, more exposure to media and advertisements, availability of left-over medication may be the influencing factors for the higher SM practice among final year students in the current study. Several studies conducted in the region have evidenced that both medical websites and advertisements play a key role in inducing the SM behaviour within undergraduates [20, 21, 24–26].

The current study has evaluated the SM behaviour of undergraduates belonging to three major state universities of Sri Lanka, out of 15 government universities in Sri Lanka, which remains as a limitation. However, a satisfactory proportion of undergraduate community has been recruited from three major universities representing all the academic fields to compensate for this limitation. Further, a recall bias could occur as the SM practices were reported by the students, which remains a general limitation in this type of studies [40]. In addition, the chronic diseases, which are often associated with SM, were not assessed within the study. However, this also has been mentioned as a limitation in similar studies [2].

Findings of the current study suggest a higher prevalence of SM among the undergraduate community in Sri Lanka, which should be considered as a critical factor in health care management. Despite strict national regulations that prohibits issue of medication without proper prescriptions, findings of this study suggests easy access to medications and therapeutic drugs in Sri Lanka from community pharmacies and other sources without supervision of a physician. Numerous countries have reported similar situations [8, 12, 24, 52], emphasizing the need for more effective supervision system and tools to regulate selling of antibiotics and other medication [24]. As highlighted by the WHO, responsible SM could reduce costs and economic burden of healthcare systems and individual citizens [60]. This requires proper knowledge and transparent regulatory system to ensure responsible use of medication and therapeutic drugs via SM [20, 24]. Therefore, properly planned health education programmes, effective tools and regulations are recommended to be implemented within Sri Lanka, to empower Sri Lankans to make well informed decisions regarding SM. Especially, such educational programmes should be combined into the general curricular of the university system of Sri Lanka, to promote responsible SM within the community.

## Conclusions

In conclusion, the findings of the current study denoted a 78% prevalence rate of SM among the studied undergraduate population, which is notably high. Approximately 52.9% of the respondents were using a single drug at a time, while 63.3% were occasionally changing the administrated dose of SM. Antipyretics for fever (73.6%), drugs for cough/runny nose (73.6%), pain killers (53.1%) and Cetrazine (47.4%) were the most consumed medications used for SM by the undergraduates. The knowledge level of undergraduates on OTC medication remained limited, while community pharmacies were found to play a major role in promoting irresponsible SM. This highlights the potential risk of undergraduate population in Sri Lanka to face ill effects of SM. Locality of students, age, field of study and the year of study were recognized as the significant risk factors associated with the SM behavior among the undergraduate students. Therefore, the health authorities of Sri Lanka should pay more attention towards health education and regulations for responsible medication selling within the country, so that the wellbeing of the community could be assured through responsible SM. Further, designing of effective tools and regulations to monitor the selling of medication at the community pharmacies, and improving the knowledge and attitudes of pharmacists are also recommended to promote responsible SM within the country.

## Data Availability

The datasets used and/or analyzed during the current study are available from the corresponding author on reasonable request.

## Abbreviations

CI: Confidence Intervals
KAPs: Knowledge, Attitudes and Practices
OTC: Over the Counter
OR: Odds Ratio
SM: Self-Medication
WHO: World Health Organization
